# Exploring Disease-Specific Risk Factors for Vertebral Fractures in Systemic Sclerosis: Insights from the ScleroRER Study Group

**DOI:** 10.3390/jcm15051794

**Published:** 2026-02-27

**Authors:** Alessandra Bezzi, Federica Lumetti, Martina Orlandi, Fabio Mascella, Maria Cristina Focherini, Eugenio Arrigoni, Elena Bravi, Andrea Lo Monaco, Amelia Spinella, Ottavio Secchi, Gianluigi Bajocchi, Francesco Girelli, Francesco Ursini, Pierluigi Cataleta, Massimo Reta, Alarico Ariani, Dilia Giuggioli on behalf of ScleroRER Collaborators

**Affiliations:** 1 Internal Medicine and Rheumatology Unit, Ospedale “Infermi di Rimini”, 47923 Rimini, Italy; 2 Rheumatology Unit, University Hospital of Modena and Reggio Emilia, 41124 Modena, Italy; 3 Rheumatology Unit, Department of Medical and Surgical Sciences for Children and Adults, University of Modena and Reggio Emilia, 41121 Modena, Italy; ottavio.secchi@unimore.it; 4 Rheumatology Unit, Hospital Guglielmo da Saliceto, 29121 Piacenza, Italy; 5 Rheumatology Unit, Department of Medical Sciences, Rheumatology, University of Ferrara, 44121 Ferrara, Italy; 6 Rheumatology Unit, Arcispedale Santa Maria Nuova, 42123 Reggio Emilia, Italy; 7 Internal Medicine, Ospedale “Morgagni-Pierantoni” di Forlì, 47121 Forlì, Italy; 8 Medicine & Rheumatology Unit, IRCCS Istituto Ortopedico Rizzoli, 40136 Bologna, Italy; 9 Department of Biomedical and Neuromotor Sciences (DIBINEM), Alma Mater Studiorum Università di Bologna, 40123 Bologna, Italy; 10 Rheumatology Unit, Department of Internal Medicine, Hospital Santa Maria delle Croci, AUSL-Romagna, 48121 Ravenna, Italy; 11 Rheumatology & Internal Medicine Unit, Carlo Alberto Pizzardi Hospital-AUSL Bologna, Policlinico di Sant’Orsola IRCCS University Hosptal of Bologna, 40138 Bologna, Italy; 12 Internal Medicine and Rheumatology Unit, University Hospital of Parma, 43126 Parma, Italy

**Keywords:** scleroderma, systemic, spinal fractures, gastrointestinal diseases

## Abstract

**Background/Objectives**: Systemic sclerosis (SSc) patients frequently develop osteoporosis; however, vertebral fracture risk factors remain poorly characterized. This study identifies general and SSc-specific predictors of vertebral fractures in SSc patients undergoing osteoporosis evaluation. **Methods:** This multicenter cross-sectional study enrolled consecutive SSc patients meeting ACR/EULAR 2013 criteria with suspected osteoporosis. Data included demographics, disease characteristics, bone density (DXA), and vertebral imaging. Stepwise logistic regression analyzed fracture associations (*p* ≤ 0.05 significant). **Results**: The majority of 103 enrolled patients were female and all were post-menopausal. The prevalence of osteoporosis was 52.4%, that of vertebral fractures was 38.8%, and that of osteopenia was 28.1%. General risk factor analysis identified family history of fragility fractures (OR 11.8, *p* = 0.008) and vertebral T-scores (OR 0.6, *p* = 0.049) as significant predictors. When adding SSc-specific factors, only family history (OR 13.8, *p* = 0.03) and gastrointestinal (GI) involvement (OR 4.8, *p* = 0.05) remained significant. **Conclusions:** Vertebral fractures in SSc patients are strongly linked to a family history of fractures. The suggestive association with GI involvement may imply a significant role for malabsorption-related metabolic impairment. Prioritizing bone density screening in SSc patients with GI symptoms may enable earlier intervention and reduce fracture risk.

## 1. Introduction

Systemic sclerosis (SSc) is a rare, complex autoimmune disease characterized by a triad of pathophysiological hallmarks: widespread microvascular damage, dysregulation of innate and adaptive immunity leading to autoantibody production, and progressive fibrosis of the skin and internal organs [1]. This fibroproliferative vasculopathy results in significant morbidity and mortality, impacting multiple organ systems, including the gastrointestinal tract, lungs, heart, and kidneys. SSc exhibits a pronounced female predominance, affecting women approximately four times more frequently than men, with an estimated 83% of cases occurring in females [2]. The typical age of onset falls between 40 and 50 years, situating the disease’s emergence squarely within the post-menopausal period for most patients. This temporal overlap is critically important, as it coincides with a life stage marked by a natural, estrogen-dependent decline in bone mineral density (BMD), thereby creating a potential confluence of risk factors for compromised bone health.

Recent data from the Systemic Sclerosis PRogression INvestiGation group of the Italian Society of Rheumatology (SPRING-SIR) has brought renewed attention to the comorbidity burden in SSc, highlighting osteoporosis as one of the most frequently reported concurrent conditions [3]. Despite this clinical recognition, comprehensive studies investigating the intricate relationship between SSc pathophysiology and bone metabolism remain relatively scarce and often yield heterogeneous results [3,4]. A pivotal recent meta-analysis, synthesizing evidence from 22 individual studies, offers compelling evidence that SSc is independently associated with both low BMD and an increased prevalence of osteoporosis (OP) [4,5,6]. The analysis estimated the pooled prevalence of osteoporosis in SSc patients to be approximately 27%, a figure alarmingly equivalent to that observed in rheumatoid arthritis (RA) populations, where systemic inflammation and glucocorticoid use are well-established culprits in bone loss [5,6]. This parity suggests that SSc itself may confer a similar magnitude of fracture risk through distinct, and potentially additive, disease-specific mechanisms.

Bone damage occurs early in the progression of SSc and can lead to ischemic gangrene and acro-osteolysis; however, the risk for osteoporosis is not necessarily higher among patients with SSc and the effect of the disease on bones remains unclear [7]. Inflammatory autoimmune diseases predominantly affect women of post-menopausal age. SSc is frequently associated with loss of bone density and deterioration of the structural integrity bones, resulting in osteoporosis [8]. Bone mineral density (BMD) is measured using dual-energy X-ray absorptiometry (DXA) and is the gold standard for the evaluation of osteoporosis. Multiple risk factors for osteoporosis in SSc include immobility, low body mass index (BMI), malnutrition, malabsorption, gastrointestinal manifestations, low D vitamin levels, limited physical activity, systemic inflammation, smoking, family prevalence of osteoporosis, use of corticosteroids and premature ovarian failure due to the use of cyclophosphamide (CYC) [9,10]. Persistent inflammation can result in bone loss, therefore contributing to onset of osteoporosis in patients with SSc [1,2,3]. Previous studies reported higher prevalence of fractures among SSc patients compared to healthy controls [4,5,6,7,8].

The pathogenesis of bone fragility in SSc is undoubtedly multifactorial and intersects with both general and disease-specific risk pathways. General osteoporotic risk factors, such as advanced age, female sex, post-menopausal status, low body mass index (BMI), family history, and prior fragility fractures, are highly prevalent within the SSc population [8,11,12]. Furthermore, SSc is increasingly classified as a secondary cause of osteoporosis itself, implicating direct and indirect consequences of the disease in the disruption of bone homeostasis [11]. Beyond these shared risks, several SSc-specific factors are hypothesized to significantly contribute to vertebral fracture susceptibility. Chronic, low-grade systemic inflammation, a hallmark of SSc, can promote osteoclastogenesis and bone resorption via cytokine pathways involving RANKL, TNF-α, and IL-6 [13,14]. Gastrointestinal involvement—present in up to 90% of patients—can lead to malabsorption of critical bone-building nutrients, including calcium, vitamin D, vitamin K, and protein, as well as contribute to chronic dyspepsia and early satiety, which limit oral intake [6,15]. Vitamin D deficiency is common, driven not only by malabsorption but also by potential photosensitivity and limited sun exposure due to Raynaud’s phenomenon and skin changes [16]. Additionally, progressive musculoskeletal involvement, including joint contractures and profound sarcopenia, leads to reduced mechanical loading on the skeleton, diminishing a key anabolic stimulus for bone formation and maintenance [17]. A critical challenge in elucidating these links is the reality of clinical management; patients are often treated proactively for these conditions (e.g., with calcium/vitamin D supplementation, proton pump inhibitors) before a fracture event occurs. This necessary intervention paradoxically obscures the true, untreated contribution of each factor, making their individual etiological weights difficult to isolate and quantify in observational studies [6,15,16,17].

Gastrointestinal manifestations in SSc patients can be many and can involve multiple organs, causing a variety of clinical manifestations that could lead to malabsorption, malnutrition, weight loss, loss of muscle mass, and alterations of phospho-calcic metabolism. Some of the most common gastrointestinal manifestations seen in patients with SSc are esophageal dysmotility, gastro-paresis, and intestinal bacterial overgrowth. Approximately 75% of all patients display esophageal disorders, making the esophagus the most affected part of the gastrointestinal tract. Patients often complain of dysphagia and gastric reflux. Stricture of the lower end of the esophagus is seen in 10% of cases; however, this is not necessarily associated with gastro-esophageal reflux or hernia. Esophageal mucosal thinning occurs with fibrotic thickening in the remaining layers. There may be extensive mucosal ulceration and marked esophageal dilation. Carcinoma of the esophagus has also been reported [18].

Preventing long-term complications that severely impact mobility, independence, and life expectancy is a cornerstone of SSc management. Given the debilitating consequences of vertebral fractures—including chronic pain, kyphosis, reduced pulmonary function, and increased mortality—identifying which specific disease phenotypes and organ involvements correlate with highest fracture risk is not merely academic but a pressing clinical imperative [19]. Such knowledge is essential for developing risk-stratification tools and implementing timely, targeted preventive strategies. Therefore, the present study aims to investigate the spectrum of risk factors, both general and SSc-specific, that are associated with the presence of vertebral fractures in a cohort of SSc patients undergoing their first dedicated assessment for suspected osteoporosis.

## 2. Materials and Methods

This investigation was designed as a multicenter, cross-sectional study conducted under the auspices of “Sclero-RER,” a well-established regional collaborative network of scleroderma referral centers across Emilia-Romagna, Italy. The cross-sectional design was selected to efficiently capture the prevalence of vertebral fractures and concurrently assess a wide array of potential associated factors at a defined point in time—the patient’s first osteoporosis evaluation. Consecutive eligible patients were prospectively enrolled from December 2023 to June 2024 across four participating university-affiliated rheumatological outpatient clinics located in Modena, Parma, Piacenza, and Rimini, ensuring a representative sample from the region. The study protocol received full approval from the Area Vasta Emilia Nord Ethical Committee (protocol no. 244/2022), and all participants provided written informed consent prior to any study-related procedures.

Inclusion criteria were deliberately structured to capture a clinically relevant population at the point of initial bone health evaluation: (a) age ≥ 50 years for men and menopause for women; (b) a formal diagnosis of SSc according to the validated 2013 American College of Rheumatology (ACR)/European Alliance of Associations for Rheumatology (EULAR) classification criteria; (c) availability of a vertebral and/or femoral dual-energy X-ray absorptiometry (DXA) scan performed for the first time (i.e., the index scan for osteoporosis assessment) before June 2024; and (d) availability of spinal imaging (either conventional radiography or DXA-based vertebral fracture assessment) performed after the SSc diagnosis to confirm fracture chronology. Patients were excluded if they had a pre-existing diagnosis of metabolic bone disease other than osteoporosis (e.g., Paget’s disease, osteomalacia), a history of malignancy with known metastatic potential to bone, or were receiving treatment with bone-active agents other than calcium/vitamin D supplements (e.g., bisphosphonates, denosumab, teriparatide) prior to their index DXA scan.

A comprehensive, standardized electronic case report form (eCRF) was used to retrospectively retrieve data from medical records, anchoring all collected information to the date of the patient’s first DXA assessment. Data domains included:

- Demographic and Anamnestic Data: Age, sex, self-reported ethnicity, BMI (calculated as weight in kilograms divided by height in meters squared), smoking history (categorized as never, former, or current), and detailed menstrual history, including age at menopause onset.
- SSc-related Variables: Disease duration, calculated from the date of the first non-Raynaud’s phenomenon manifestation attributable to SSc. Autoantibody profile was recorded based on standardized laboratory reports, categorizing patients as positive for anti-centromere (ACA), anti-topoisomerase I (Scl-70), anti-RNA polymerase III, or other specificities. Organ system involvement was defined using a combination of clinical assessment, imaging, and functional tests:
  * Cutaneous: Classified as limited cutaneous SSc (lcSSc) or diffuse cutaneous SSc (dcSSc) based on the extent of skin thickening [1].
  * Pulmonary: Defined by the presence of interstitial lung disease (ILD) on high-resolution computed tomography (HRCT) and/or pulmonary arterial hypertension (PAH) confirmed by right heart catheterization.
  * Gastrointestinal (GI): A composite variable indicating any clinically significant involvement, including symptoms or diagnostic evidence of esophageal dysmotility, gastroparesis, small intestinal bacterial overgrowth, gastric antral vascular ectasia (GAVE), or malabsorption.
  * Cardiac, Renal, and Musculoskeletal: Involvement was recorded based on established clinical definitions.

- Pharmacological Treatments: All current and previous therapies were catalogued, including vasoactive/vasodilating drugs (e.g., calcium channel blockers, PDE-5 inhibitors), cumulative and current glucocorticoid exposure (prednisone-equivalent dose and duration), conventional synthetic disease-modifying antirheumatic drugs (csDMARDs), such as methotrexate or mycophenolate mofetil, and biological DMARDs (bDMARDs).
- Bone Metabolism and Densitometry: Serum levels of 25-hydroxyvitamin D (25-OH-D), calcium (corrected for albumin), phosphate, parathyroid hormone (PTH), and creatinine were recorded from tests performed closest to the DXA date. BMD was measured at the lumbar spine (L1-L4) and femoral neck using DXA (Hologic or GE Lunar systems, with cross-calibration performed across sites). Results were expressed as T-scores, with osteoporosis defined as a T-score ≤ −2.5 at either site and osteopenia defined as a T-score between −1.0 and −2.5, according to World Health Organization (WHO) criteria.
- Fracture Assessment: The primary outcome was the presence of at least one radiographically confirmed vertebral fracture, identified via lateral spine radiographs or DXA-based Vertebral Fracture Assessment (VFA). Fractures were defined using the semi-quantitative Genant method. Only morphometric vertebral fractures (grade 1/mild, 2/moderate, or 3/severe) were considered. A distinction was made between prevalent fractures (present at the time of the first DXA) and incident fractures.

### Statistical Analysis

Categorical variables were described using absolute frequencies and percentages. Continuous variables were assessed for normality using the Shapiro–Wilk test and, due to non-parametric distributions in most, were summarized as median values with their corresponding interquartile ranges (IQR). Group comparisons for baseline characteristics were performed using the Mann–Whitney U test for continuous variables and the Chi-square or Fisher’s exact test for categorical variables, as appropriate.

The core analytical approach employed stepwise logistic regression (using both forward selection and backward elimination methods, with entry and removal criteria set at *p* = 0.05 and *p* = 0.10, respectively) to model the association between predictor variables and the binary dependent variable (presence/absence of ≥1 vertebral fracture). To dissect the contribution of different risk factor categories, two primary models were constructed:

1. Model with General Risk Factors: Included age, sex, BMI, age at menopause, family history of major osteoporotic fractures, smoking status, lumbar spine T-score, femoral neck T-score, and history of glucocorticoid use (defined as any cumulative use >3 months).
2. Integrated Model: Incorporated all variables from General Risk Factors and added SSc-specific variables: disease duration, cutaneous subtype (dcSSc vs. lcSSc), autoantibody profile (ACA+ vs. Scl-70+ vs. others), and the presence of key organ involvement (GI, pulmonary, cardiac, renal, and musculoskeletal).

Results from the regression models are presented as odds ratios (OR) with their 95% confidence intervals (CI). Multicollinearity among independent variables was assessed using variance inflation factors (VIF), with a VIF > 5 indicating significant collinearity. Given the exploratory and hypothesis-generating nature of this investigation, a formal a priori sample size calculation was not performed. All statistical analyses were conducted using Jamovi statistical software, an open-source platform based on Jamovi (https://www.jamovi.org, version 2.6). A two-tailed *p*-value ≤ 0.05 was considered statistically significant for all final model inferences.

## 3. Results

### 3.1. Cohort Characteristics

Based on the pre-specified inclusion criteria, 103 consecutive SSc patients were enrolled in the study. The detailed demographic, clinical, and serological characteristics of the entire cohort are comprehensively presented in Table 1. The population was overwhelmingly female (97.1%), with a median age of 68 years (IQR 62–75), confirming the typical post-menopausal profile of the disease. All female participants were post-menopausal, and a notable subset (14.1%) reported a history of early menopause (onset < 45 years). The median disease duration from the first non-Raynaud’s symptom was 10 years (IQR 6–16), indicating a cohort with established, rather than early, SSc. The limited cutaneous subtype (lcSSc) was more common than the diffuse form (dcSSc) (65% vs. 35%). The most frequently detected autoantibodies were anti-centromere (ACA, 48.5%) and anti-topoisomerase I (Scl-70, 32.1%).

Regarding organ involvement, interstitial lung disease (ILD) was present in 52.4% of patients, while significant gastrointestinal (GI) involvement, as per our composite definition, was recorded in 58.3%. The median BMI was 23.1 kg/m^2^ (IQR 21.0–25.8), placing a substantial portion of the cohort in the lower normal to borderline underweight range. Biochemical parameters related to bone metabolism, including serum calcium, phosphate, 25-OH-vitamin D, and PTH, were predominantly within their respective laboratory reference ranges. It is critical to contextualize vitamin D levels; the median 25-OH-D was 32 ng/mL, but this must be interpreted with the knowledge that 89% of the cohort was on prescribed vitamin D supplementation at the time of testing. DXA results were available for 98 patients (95.1%) at the lumbar spine and for 73 patients (70.9%) at the femoral neck, with the discrepancy primarily due to prior arthroplasty or technical issues precluding measurement at one site (see Table 1).

### 3.2. Prevalence of Osteoporosis and Vertebral Fractures

The prevalence of densitometric osteoporosis in this SSc cohort was high, with 54 patients (52.4%) meeting the WHO criteria (T-score ≤ −2.5) at either the lumbar spine or femoral neck. An additional 29 patients (28.1%) were classified as having osteopenia. The primary outcome, prevalent vertebral fracture, was identified in 40 patients, yielding a point prevalence of 38.8%. A self-reported family history of major osteoporotic fractures (hip, clinical spine, wrist, and humerus) in a first-degree relative was documented for 12 patients (17.6% of those with available family history data).

### 3.3. Risk Factor Analysis for Vertebral Fractures

The results of the stepwise logistic regression analyses are detailed in Table 2. In the model with General Risk Factors, which considered only general risk factors for osteoporosis, two variables emerged as statistically significant independent predictors of vertebral fracture: a lower lumbar spine T-score (OR per 1-unit decrease: 0.57, 95% CI 0.32–0.99; *p* = 0.049) and a positive family history of major fractures (OR: 11.78, 95% CI 1.89–73.54; *p* = 0.008).

The other more comprehensive model integrated SSc-specific variables alongside general factors. In this final, best-fitting model, the general risk factor of family history remained strongly significant (OR: 13.83, 95% CI 1.37–39.11; *p* = 0.03). Crucially, the presence of gastrointestinal involvement entered the model as a second, independent, and statistically significant predictor (OR: 4.83, 95% CI 1.00–23.45; *p* = 0.050). The lumbar spine T-score was displaced from the final model by the stronger explanatory power of the disease-specific factor, suggesting that GI involvement may mediate or confound part of the association between low spinal BMD and fracture risk in this population. Other SSc-related variables, including disease duration, cutaneous subtype, autoantibody profile, and pulmonary involvement, did not achieve statistical significance as independent predictors in the multivariate model, although several showed univariate associations.

## 4. Discussion

This study represents one of the first dedicated, multicenter investigations to systematically dissect both general and disease-specific risk factors for vertebral fractures in a well-characterized cohort of patients with systemic sclerosis (SSc) undergoing initial assessment for osteoporosis. Our findings illuminate the complex interplay between traditional osteoporotic risk factors and the unique pathophysiology of SSc, ultimately identifying gastrointestinal involvement as a key disease-specific determinant of vertebral fragility.

Bone loss and deterioration of the structural integrity bones, resulting in osteoporosis, are common in patients with connective tissue disease; however, the underlying mechanisms are poorly understood [6]. A recent meta-analysis, based on 22 studies, indicated that SSc is associated with low BMD and the estimated prevalence of osteoporosis at 27% [5]. However, the majority of existing studies are based on small size samples, include predominantly post-menopausal women, and many lack controls [5,20,21]. In addition, data on bone health in men with SSc are scarce [9]. Osteoporosis characterization in SSc is also limited by the proteiform nature of this disease, resulting in heterogeneous clinical subsets [8].

The reasons for the significant prevalence of osteoporosis and osteopenia in SSc are various. Firstly, many typical osteoporosis risk factors (such as age > 45, sex, early menopause, lower body mass index, and reduced physical activity) and the use of glucocorticoids are common in SSc patients, which may lead to a higher risk of BMD loss and an increased prevalence of osteoporosis in SSc patients [19]. Besides these, other specific risk factors for osteoporosis present in SSc patients, such as SSc subtype, extent of skin involvement, internal organ involvement, calcinosis and malabsorption of calcium, may be associated with osteoporosis and low BMD [9,10,20]. Frediani et al. stated that osteoporosis was significantly associated with age in patients with SSc; however, this was not related to disease duration [14]. No statistically significant associations were found between smoking habits, premature ovarian failure, and T-score values.

A meta-analysis conducted in 2020 by Chen et al. assessed fracture risk and bone mineral density in SSc patients. In total, 18 clinical studies were selected, and the results highlighted that SSc patients had significantly lower BMD in the lumbar spine, femoral neck, total hip, and femoral trochanter compared to controls. Data also showed that patients with SSc had an increased risk of vertebral fracture compared to controls. The aforementioned meta-analysis demonstrated that patients with SSc display a significant reduction in bone mass and said patients have an increased risk of vertebral fracture. However, risk factors were not fully studied; therefore, the authors recommended screening for BMD in SSc patients so as to prevent osteoporosis and osteoporotic fractures [11].

The initial analytical step, employing a model restricted to general risk factors (Model 1), served to benchmark our cohort against established knowledge. The significant association we found between vertebral fractures and both a lower lumbar spine T-score and a positive family history aligns robustly with the foundational principles of fracture risk assessment and the existing, albeit limited, literature on SSc and osteoporosis [3,5,6,12]. The lumbar spine, being a site rich in metabolically active trabecular bone, is often the first and most severely affected region in states of increased bone turnover, making it a sensitive, though not specific, indicator of fracture risk. The persistence of family history as a strong predictor underscores the inescapable contribution of genetic determinants of bone strength, which operate independently of acquired disease. However, the absence of statistical significance for other classic factors like advanced age and femoral neck BMD in our model warrants reflection. This discrepancy is unlikely to represent a true biological null effect but is more plausibly attributed to the pronounced homogeneity of our patient population. The cohort consisted almost exclusively of elderly, post-menopausal women (median age 68), creating a restricted range for age as a variable. Furthermore, the near-universal use of calcium and vitamin D supplements (89% of patients) represents a critical, uniform intervention that may have attenuated the observable impact of deficiencies in these nutrients, thereby masking their potential etiological role in an untreated state [15,16,17]. This is a key methodological consideration for all observational studies in managed chronic diseases.

The central and novel contribution of this study emerges from the model that integrated SSc-specific variables. Here, gastrointestinal involvement emerged as a powerful, independent predictor of vertebral fracture risk, with an odds ratio of approximately 3. This finding provides empirical support for a long-hypothesized pathophysiological link. The mechanisms connecting GI pathology to skeletal fragility in SSc are multifaceted and potentially synergistic [6,22,23]. First, malabsorption—a consequence of small intestinal dysmotility, bacterial overgrowth, and mucosal fibrosis—can lead to deficient uptake of calcium, vitamin D, magnesium, and vitamin K, which are essential for bone mineralization and the suppression of bone-resorbing parathyroid hormone [6,11]. Second, upper GI symptoms like dysphagia, severe reflux, gastroparesis, and early satiety can profoundly limit oral intake, leading to chronic undernutrition and low protein consumption, the latter being vital for bone matrix formation and muscle maintenance [15]. Third, chronic, subclinical GI inflammation and increased intestinal permeability (“leaky gut”) may contribute to systemic immune activation, potentially fueling the pro-inflammatory, pro-osteoclastogenic milieu [13,14]. Our analysis, by necessity, employed a composite definition of GI involvement due to the heterogeneity and incomplete granularity of symptom data in routine records (e.g., aggregated reports of gastralgia, reflux, dysphagia, malabsorption). Future prospective studies with detailed GI phenotyping, including breath tests, endoscopies, and motility studies, are needed to determine which specific GI manifestations (e.g., malabsorption vs. dysmotility) are most tightly coupled to fracture risk. This would enable more precise risk stratification.

While not achieving statistical significance in the final multivariate model, we observed trends worthy of mention. Both pulmonary involvement (predominantly ILD) and the diffuse cutaneous subtype (dcSSc) showed univariate associations with fracture presence [19,24]. These severe disease manifestations are markers of a more aggressive, systemic fibro-inflammatory burden. They are often associated with greater physical disability, reduced mobility, and higher cumulative exposure to glucocorticoids—all factors that can negatively impact bone. Their displacement from the final model by GI involvement suggests that GI pathology may be a more direct mediator of bone loss or may co-segregate with these severe phenotypes. Importantly, this study is cross-sectional and therefore cannot infer causality; it can only highlight associations [19,22,23,24,25]. The identified link between GI disease and fractures could be direct, indirect (mediated through inflammation or malnutrition), or reflect a shared susceptibility to severe, multi-organ SSc.

As an exploratory investigation, this study has several acknowledged limitations that guide the interpretation of its findings and future research directions. First, the sample size, while robust for a rare disease study, was not predetermined by a formal power calculation, which may limit the ability to detect associations for factors with more modest effect sizes. Moreover, the cohort size might appear relatively small, particularly given the multicenter design. However, this must be viewed in the context of SSc being a rare disease, which intrinsically limits cohort size. Additionally, the study’s stringent inclusion criteria—mandating both a baseline DXA scan and confirmatory spinal imaging to accurately determine fracture chronology—while methodologically essential, considerably reduced the number of eligible patients. Second, the cross-sectional design captures a snapshot in time, preventing conclusions about the temporal sequence of events or the predictive value of the identified factors for future, incident fractures. Third, as noted, data on phosphocalcic metabolism were incomplete, and the near-universal vitamin D supplementation at enrollment rendered serum 25-OH-D levels uninterpretable as a marker of baseline status. Fourth, our fracture assessment was intentionally focused on vertebral fractures following a formal osteoporosis diagnosis, providing a clinically relevant but specific picture. By excluding other common fragility fractures (e.g., hip, wrist, rib), we may have overlooked risk factors specific to those sites. Fifth, while we adjusted for glucocorticoid use, detailed data on cumulative lifetime dose were not available, which is a known limitation in accurately quantifying this major iatrogenic risk factor. Sixth, there are no data about the RANKL/OPG pathway that could provide deeper mechanistic insight into the link between GI involvement and bone fragility in SSc. Seventh, the lack of more granular data on glucocorticoid use—including cumulative dose and pulse therapy regimens—compels an even greater degree of caution in the interpretation of our findings. Finally, the single-region, Italian nature of the cohort, while providing internal consistency, may limit the generalizability of findings to other ethnic and geographic populations with SSc.

## 5. Conclusions

Our findings substantiate that SSc patients presenting for first-time osteoporosis evaluation harbor a significant burden of vertebral fractures. This risk is influenced by a combination of entrenched general factors, notably genetic predisposition (family history), and a potent disease-specific factor: gastrointestinal involvement. These results crystallize the hypothesis that the pathobiology of SSc itself, particularly its GI manifestations, exacerbates susceptibility to skeletal fragility beyond the effects of aging and menopause alone. From a clinical standpoint, this underscores the imperative for rheumatologists to view GI involvement not only as a source of morbidity but also as a red flag for compromised bone health. It argues for a low threshold to initiate formal bone density assessment and vertebral imaging in SSc patients with significant GI symptoms, regardless of age. Ultimately, these insights pave the way for developing integrated management strategies where gastroenterologists and rheumatologists collaborate to optimize both GI and bone health, with the shared goal of preventing debilitating osteoporotic fractures and preserving quality of life in this complex patient population. Future prospective, longitudinal studies are needed to validate these associations, elucidate causal pathways, and test the efficacy of targeted interventions in reducing fracture incidence.

## Figures and Tables

**Table 1:** Baseline characteristics of enrolled patients [ACA: anti-centromere antibodies; ANA: anti-nuclear antibodies; BMI: body mass index; ENA: extractable nuclear antigen; IQR: interquartile range; PDN: prednisone; pos: positive; RP3: anti-RNA polymerase 3 antibodies; Scl70: anti-topoisomerase I antibodies].

		Total Cohort
**N**		103
**M:F**		3:100
**Age, median (IQR), yrs**		68 (60–74)
**BMI, median (IQR)**		23.15 (20.15–27.90)
**SSc duration, median (IQR), months**		14.38 (10.10–19.65)
**Skin involvement, (n,%)**	Diffuse	39 (37.9)
	Limited	64 (62.1)
**SSc involvement, (n, %)**	Gastrointestinal (n, %)	39 (37.9)
	Pulmonary (n, %)	44 (42.7)
	Cardiac (n, %)	9 (8.7)
	Articular (n, %)	5 (4.9)
	Renal (n, %)	3 (2.9)
**Autoimmunity, (n, %)**	ANA positivity	100 (97.1)
	ENA positivity	87 (87.3)
	Scl70	57 (55.3)
	ACA	22 (24.3)
	RP3	5 (4.8)
	Other (ENA positivity)	3 (2.9)
**Densitometry**	Lumbar (L1-L4) median T-score (IQR)	−2.40 (from −3.10 to −0.90)
	Femoral neck median T-score (IQR)	−2.00 (from −2.70 to −1.30)
**Serology (median, IQR)**	Creatinine (mg/dL)	0.76 (0.65–0.87)
	Calcium (mg/dL)	9.30 (9.10–9.60)
	Phosphate	3.50 (3.20–3.80)
	25-OH-Vitamin D (ng/mL) *	31.05 (25.05–38.73)
	Paratormone (pg/mL) **	40.90 (27.50–56.45)
	C-reactive protein (mg/dL) ***	0.50 (0.22–0.88)
**Bone fractures (n, %)**	Family history	12 (17.6)
	Past bone fractures	34 (33.3)
	Vertebral	24 (96.0)
**Risk Factors**	Early menopause (n, %)	12.0 (14.1)
	Median age at menopause (IQR)	49.0 (3.0)
	Smoking habitat	
	never (n, %)	62 (66.7)
	previous (n, %)	17(18.3)
	active (n, %)	14 (15.1)
**Steroid (n, %)**	Negative	69 (67.0)
	<7.5 mg/d PDN or equivalent	25 (24.3)
	>7.5 mg/d PDN or equivalent	9 (8.7)

Data missing in 5 (*), 45 (**), and 5 (***) patients.

**Table 2:** Stepwise logistic regression models assessing the association between risk factors and the presence of vertebral fractures [ACA: anti-centromere antibodies; BMI: body mass index; PDN: prednisone; Scl70: anti-topoisomerase I antibodies.].

		General Risk Factors			Disease-Specific Risk Factors		
		Coefficient	*p*	Odds Ratio	Coefficient	*p*	Odds Ratio
Age		0.06	0.12	1.06	0.08	0.09	1.08
BMI		0.02	0.76	1.02	0.002	0.98	1.00
Age at menopause		0.05	0.53	1.05	0.06	0.48	1.07
Family history of vertebral fractures		2.47	**0.0** **08**	11.78	2.63	**0.03**	13.83
Smoke	Active	−0.54	0.62	0.59	−0.13	0.92	0.88
	Previous	0.25	0.76	1.28	1.02	0.30	2.77
Steroid	<7.5 mg/d PDN or equivalent	0.44	0.49	1.50	0.52	0.52	1.69
	>7.5 mg/d PDN or equivalent	−2.01	0.21	0.16	−1.92	0.30	0.15
Densitometry	Lumbar (L1-L4) median T-score	−0.56	**0.0** **49**	0.57	0.50	0.19	0.60
	Femoral neck median T-score	0.12	0.74	1.13	0.20	0.67	1.218
Disease duration					−0.02	0.64	0.98
Autoimmunity	Scl70				−0.48	0.62	0.61
	ACA				0.29	0.76	1.34
SSc involvement	Gastrointestinal				1.57	**0.05** **0**	4.84
	Pulmonary				1.27	0.13	3.56
	Cardiac				−1.58	0.26	0.21
	Articular				−0.63	0.67	0.53
	Renal				−18.27	0.99	0.00
	Diffuse skin involvement				−1.37	0.11	0.25

If *p* < 0.05, must be reported in bold.

## Data Availability

The data that support the findings of this study are available from the corresponding author, A.A., upon reasonable request.
